# Comparing circular and flexibly-shaped scan statistics for disease clustering detection

**DOI:** 10.3389/fpubh.2024.1432645

**Published:** 2025-01-08

**Authors:** Lina Wang, Xiang Li, Zhengbin Zhang, Haoxun Yuan, Pengfei Lu, Yaru Li

**Affiliations:** ^1^School of Computer Science and Technology, Zhengzhou University of Light Industry, Zhengzhou, China; ^2^Institute of Surveying and Mapping, Information Engineering University, Zhengzhou, China; ^3^Tuberculosis Prevention and Control Office, Wuhan Institute for Tuberculosis Control, Wuhan, China

**Keywords:** spatial scan statistics, disease cluster detection, SaTScan, FleXScan, Gini coefficient, log-likelihood ratio (LLR), cluster size

## Abstract

The accuracy of spatial clustering detection is crucial for public health policy development and identifying etiological clues. Circular and flexibly-shaped scan statistics are widely used for disease cluster detection, but differences in results arise mainly due to parameter sensitivity and variations in the scanning window shapes. This study aims to analyze the impact of parameter settings on the results of these methods and compare their performance in disease clustering detection. Using tuberculosis data from Wuhan, China (2015–2019), the study identified the optimal parameter settings—MSWS and *K*-value—for each method to ensure accurate clustering. A comprehensive comparison was made using two quantitative indicators, the LLR value and cluster size, as well as clustering visualizations. The results show that the optimal MSWS parameter for SaTScan is determined through a Gini coefficient-based stepwise-threshold-reduction approach, while a *K*-value of 30 is ideal for FleXScan. SaTScan tends to produce more regular clusters, while FleXScan often generates more irregular clusters. FleXScan detects fewer clusters but with higher LLR values and larger average cluster sizes, although the maximum cluster size is smaller. These findings provide valuable insights for optimizing disease clustering detection methods and enhancing public health interventions.

## 1 Introduction

The purpose of spatial cluster detection of diseases is to identify whether clustering disease exists and to locate the areas where these clusters occur. This information can provide clues for further etiological investigation. Spatial scan statistics have been widely used as a technique for detecting disease clusters (1–4). This method was first introduced by Kulldorff, along with the freely available SaTScan software, and has since been extended with several different statistical models. The method utilizes a likelihood ratio test statistic to evaluate a large number of different and overlapping scanning windows. The test statistic is formulated based on a probability model depending on the data type, such as the Poisson model for count data. However, this method is limited to circular scan windows for detecting compact clusters, which may struggle to accurately identify non-circular clusters. Consequently, other researchers have proposed alternative approaches that employ different scanning window selection schemes, such as elliptical (5–7) and flexibly-shaped windows (8–10).

A popular alternative for detecting clusters with arbitrary shapes is the flexibly shaped spatial scan statistic proposed by Tango and Takahashi, which is implemented in the FleXScan software. This method employs an adjacency expansion search, scanning adjacent units in the spatial region to detect irregularly shaped clusters (8–10). However, the selection of scanning windows in FleXScan is dependent on an exhaustive search strategy, which leads to exponential runtime scaling as the *K*-value increases. Here, the *K-*value is a constant that indicates the maximum number of sub-regions allowed within a preset window, severing as a crucial parameter in the implementation of the FleXScan method. Due to computational constraints, the *K*-value is typically limited to 30, with a default of 15. To address these limitations, Tango and Takahashi (10) proposed a restricted version of the flexibly-shaped scan statistic that focuses exclusively on regions with elevated risk. This modified approach offers improved computational efficiency and enables the use of a *K-*value up to 30 (10, 11).

In addition, Speakman et al. (12) developed the GraphScan method for detecting connected clusters of arbitrary shapes in graph or network data. This method improves search efficiency by incorporating a branch-and-bound depth-first search approach, which enhances the brute force algorithm used in FleXScan. Cadena et al. (13) presented a framework for network anomaly detection based on scan statistics that outperforms existing methods in terms of performance and scalability. Meysami M et al. (6) proposed the flexible–elliptical scan method, which combines the flexible and elliptic scan methods to address their respective limitations and leverage their advantages. However, for non-statistical users such as epidemiologists and public health researchers, user-friendly software may be more practical than introducing new algorithms. Currently, the most commonly used methods are still circular and flexibly-shaped scan statistics, which can be directly implemented in SaTScan and FleXScan, respectively.

However, both methods face the challenge of parameter setting during implementation, as the cluster results are highly sensitive to these parameters. For example, the performance of circular spatial scan statistics is influenced by the selection of the maximum scanning window size (MSWS) (14–16). If the MSWS is too large, the detected clusters may be overly large and may include areas with non-elevated risk. Conversely, if it is too small, numerous small clusters may be detected. Different MSWS values yield varying cluster sizes, locations, and numbers within the same dataset. Although it is common to use 50% of the total population as the default setting for MSWS in SaTScan, this may result in an overly large cluster. Therefore, determining the optimal MSWS value is crucial for the SaTScan method. Performance indicators such as sensitivity, specificity, positive predictive value (PPV), and Youden’s index (YDI) are typically used to select the optimal MSWS (16), but these metrics are often only available in simulation studies. Han et al. (17) proposed the Gini coefficient as an effective criterion for determining optimal cluster reporting sizes, which helps avoid unnecessarily large and less informative clusters. This approach has been implemented in SaTScan version 9.3 and has shown success with both simulated and real data (18, 19). Another indicator, called the maximum clustering heterogeneous set-proportion (MCHS-P), was introduced by Wang et al. (16) for selecting suitable MSWS. However, the Gini coefficient remains widely used due to its direct application through SaTScan, despite some limitations pointed out by Li et al. (15) and Wang et al. (16).

The FleXScan method suggests that setting *K* = 30 theoretically helps achieve the optimal maximum likelihood clustering (MLC). However, it is important to understand the impact of different *K*-values on the final clustering results. For example, if we set *K* = 15, can we still achieve a good MLC, and what are the differences between the two clustering results? Evaluating the influence of *K*-value requires practical analysis and comparison.

The accuracy of spatial cluster detection results is of great significance for the formulation of prevention and control policies in the region and the detection of further etiology. Spurious cluster results, however, may have unnecessary negative impacts on the socio-economic development of that region (20). Therefore, selecting the appropriate parameter settings is important for accurate cluster identification. Unfortunately, there is currently no standard reference criterion for parameter selection.

Previous studies have demonstrated that different research purposes require different parameter combinations for analysis. However, most previous studies are based on simulated data with specific assumptions, and the conclusions drawn may not be fully applicable to real data, which has certain limitations. The optimal parameter combination varies with different data, and the conclusions of simulation research are often difficult to extend to more complex and variable real-world scenarios without sufficient prior knowledge.

Therefore, the purpose of our study is to compare the differences between the two different scanning window methods, and to determine the optimal parameter settings for each method. This will clarify the impact of parameter settings on the results and provide a reference for other researchers. We will utilize real pulmonary tuberculosis disease data (at the township level) from Wuhan, spanning 2015 to 2019, as our research dataset. We will provide optimal parameter settings for the two scanning window types in different years and compare the spatial clustering results obtained from these methods.

## 2 Study area and data

**Figure 1:**
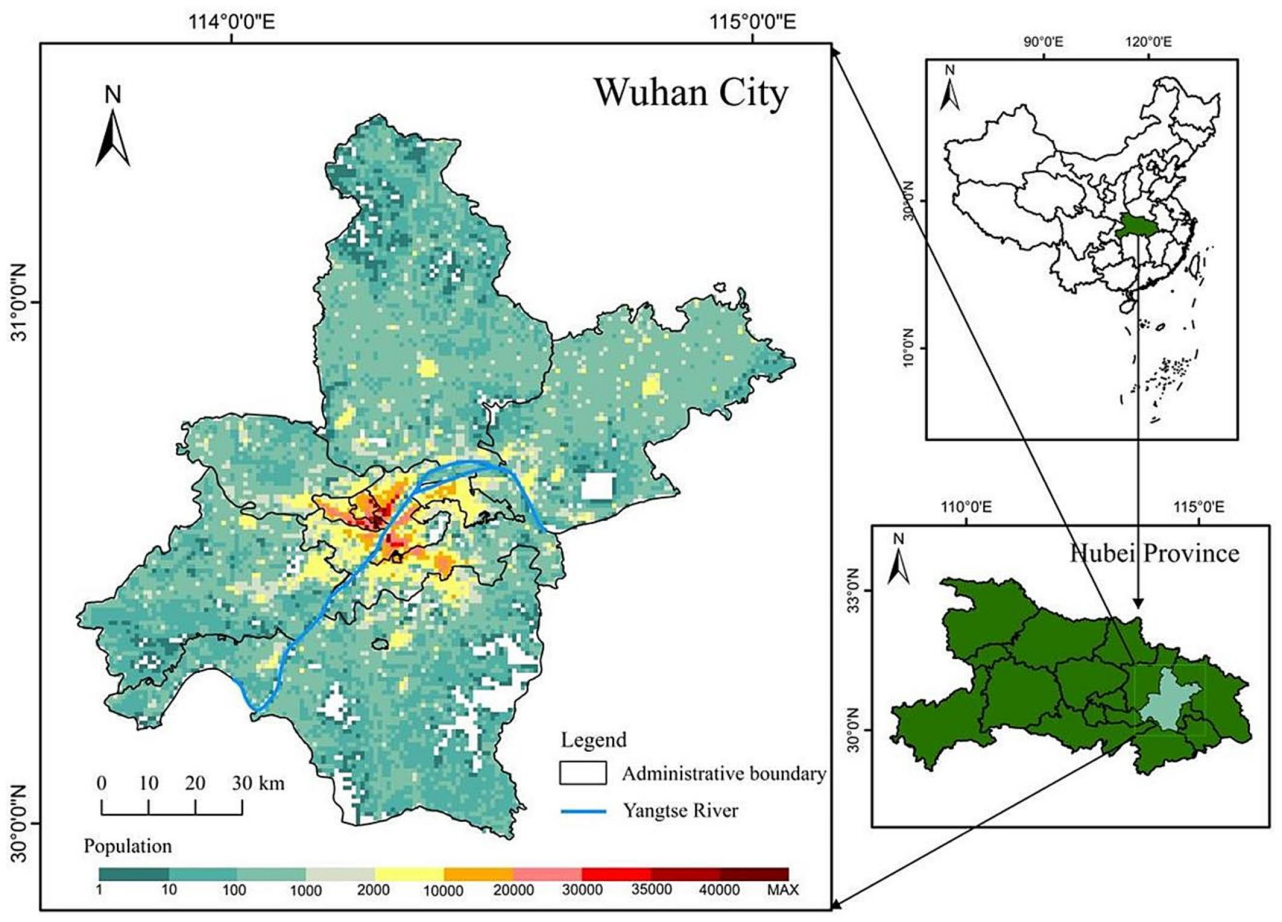
Study area.

Our study area is Wuhan City, the capital of Hubei Province, located in central China. Known as “the River City,” Wuhan is situated at the confluence of the Yangtze River and the Han River the largest tributary of the Yangtze. This strategic location has made Wuhan a crucial transportation hub, connecting various parts of China through its extensive network of railways, highways, and waterways. Wuhan City comprises 13 county-level units and 164 town-level units, with a total area of 8,569.15 square kilometers. As of the end of 2021, according to official information, Wuhan had a permanent population of 12.3265 million, with its population spatial distribution shown in Figure 1. It can be observed from the figure that the central urban area is densely populated, while the peripheral rural areas are sparsely populated.

The case information for pulmonary tuberculosis in this study was obtained from the National Tuberculosis Management Information System, specifically the registered and managed medical records of pulmonary tuberculosis patients based on their initial diagnosis locations from 2015 to 2019. A total of 30,486 pulmonary tuberculosis patients were included in the study. We first employed geocoding techniques to spatially encode the addresses of the cases and then combined this with population demographic data to obtain the incidence rate at the township level. Thus, the final research data used in this study consisted of the pulmonary tuberculosis incidence ratesin Wuhan at the township level from 2015 to 2019.

## 3 Methods

The study involved determining optimal parameters, visualizing incidence and disease clustering results, and conducting a comparative analysis. We assessed and compared the performance of both methods in detecting disease clusters by evaluating LLR values and cluster size. To facilitate comparability, both methods were implemented using the same statistical model, specifically the Poisson statistical model.

### 3.1 Evaluation metrics for comparison

#### 3.1.1 The LLR value

The LLR value quantifies the deviation of observed data from random spatial distribution. A higher LLR value suggests a higher likelihood of non-random clustering, indicating the presence of genuine spatial clusters. Comparing LLR values allows us to assess the strength and significance of detected clusters, helping to identify meaningful and informative clusters in the analysis.

#### 3.1.2 The cluster size

The cluster size represents the number of sub-regions contained within a cluster. Restricting the cluster size may help reduce the likelihood of misclassifying random noise as clusters. Tango et al. (8) pointed out that it is unlikely for the size of a true cluster to be larger than 10–15 percent of the total number of regions. However, this is not a fixed rule and may vary depending on the specific research field and data characteristics.

### 3.2 MSWS settings for the circular scan statistic

Initially, we attempted to use the same MSWS value for different years within the same spatial region. However, this approach proved to be unreasonable, as the spatial distribution of diseases varied significantly across different years. To address this issue, we utilized Gini coefficients to assist in identifying the optimal clusters. The Gini coefficient is a statistical measure of data inequality, which helps evaluate the quality of clustering results under different MSWS values. A higher Gini coefficient indicates a more uneven spatial distribution of the clustering result, suggesting that the clustering results achieved at that particular MSWS value possess greater distinctiveness and significance in terms of differentiation.

In this study, we tested MSWS values of 5, 10, 15, 20, 25, 30, 40, and 50% for each year from 2015 to 2019.We then calculated the corresponding Gini coefficients for each MSWS value. The MSWS value associated with the highest Gini coefficient was selected as the optimal choice. Our analysis revealed that even within the same spatial region, the optimal MSWS values varied due to differences in the spatial distribution of diseases across different years.

**Figure 2:**
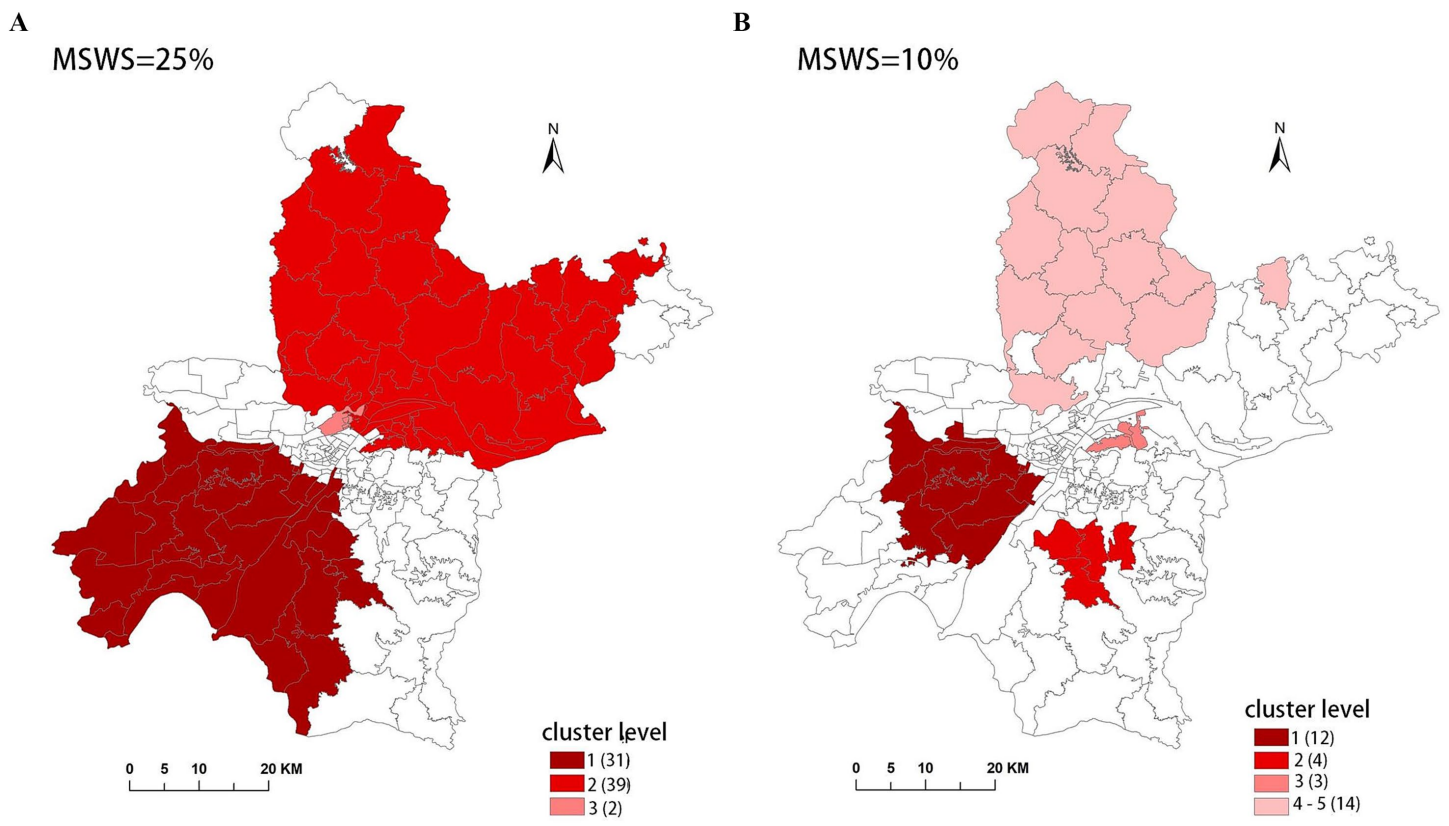
Clusters detected with different MSWS value in SaTScan. **(A)** MSWS is 25%, **(B)** MSWS is 10%.

However, we have found in practice that simply using the Gini coefficient as a criterion for determining MSWS is insufficient. According to the Gini coefficient, the optimal MSWS in 2018 should be 25%, However, the spatial clustering result at this value includes too many sub-regions, with the most likely cluster (MLC) containing 31 sub-regions and the secondary cluster containing 39 sub-regions. Together these two clusters cover nearly 50% of the total number of sub-regions. Clearly, the clusters are too large and may contain non-clustered areas. Therefore, we predetermined the MSWS value to be 10% when the Gini coefficient was the second largest. At this MSWS value, the MLC is divided into two small clusters and non-cluster regions, resulting in a significant reduction in the number of intra-cluster sub-regions. Although the LLR value of the MLC decreased, the LLR values of other clusters increased. The results are presented in Table 1 and Figure 2.

**Table 1:** Comparison of cluster results with different MSWS values in SaTScan.

MSWS = 25% (2018)						MSWS = 10% (2018)					
Level of clusters	Number of Sun-region	Number of cases	Population in risk	LLR	*p* - value	Level of clusters	Number of Sun-region	Number of cases	Population in risk	LLR	*p* - value
MLC	31	1,270	1,861,018	38.70	<0.001	MLC	12	614	815,615	32.70	<0.001
2	39	1,517	2,398,624	22.66	<0.001	2	4	242	279,704	23.58	<0.001
3	2	143	181,846	9.00	<0.001	3	3	111	111,182	17.33	<0.001
						4	1	69	60,229	15.43	<0.001
						5	13	586	895,986	10.76	0.002

Finally, considering both the Gini coefficient and the number of sub-regions included in the clusters, we determined the optimal MSWS values for this study, as shown in Table 2.

**Table 2:** The selected optimal MSWS value in different years.

Year	2015	2016	2017	2018	2019
MSWS	15%	10%	20%	10%	15%

### 3.3 *K*-value setting for the flexibly-shaped scan statistic

**Figure 3:**
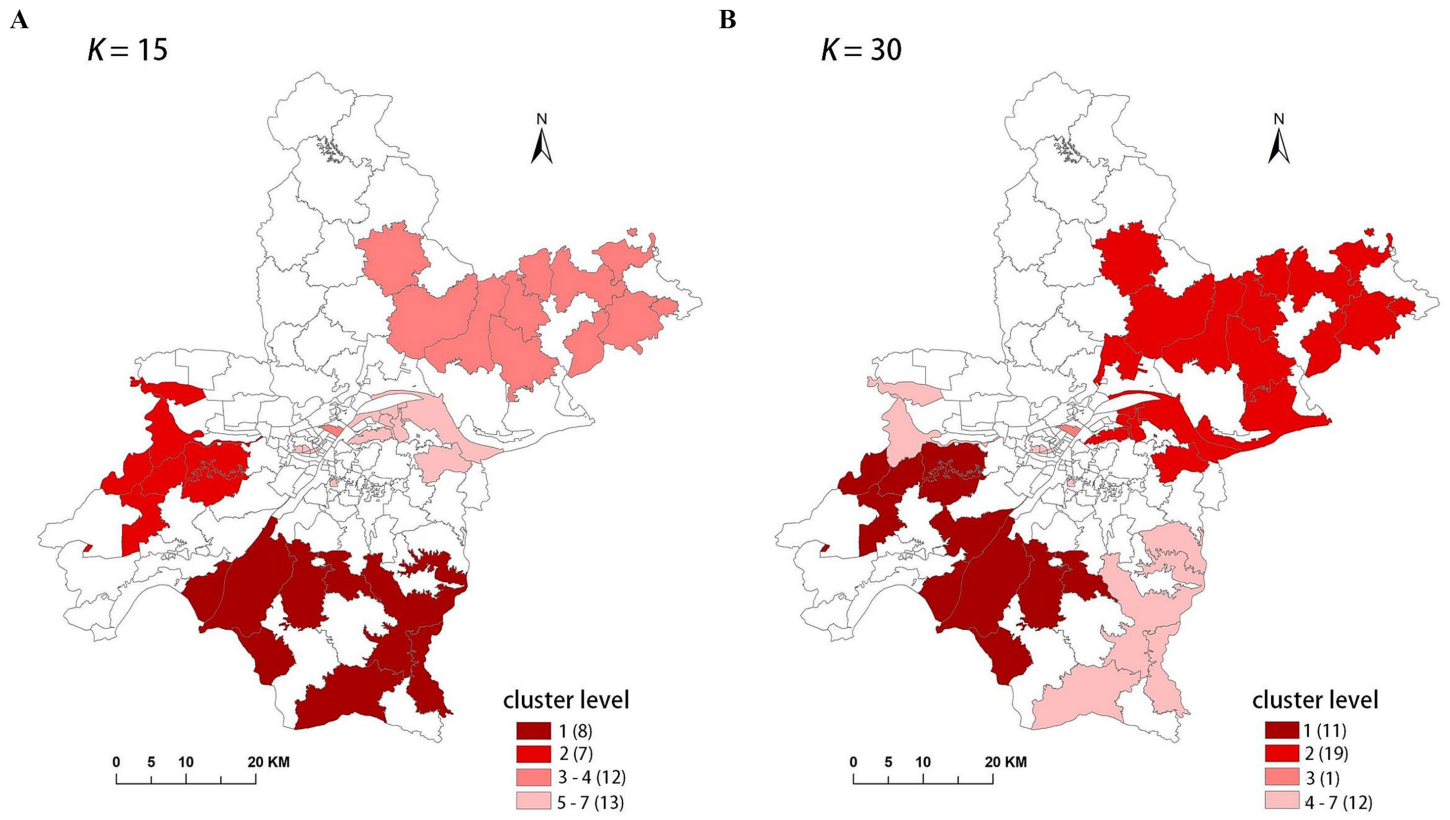
The clusters detected with different K-value in FleXScan. **(A)** *K* = 15, **(B)** *K* = 30.

Theoretically, a larger *K*-value increases the number of candidate scan windows that need to be calculated, but it also enhances the likelihood of identifying clusters with higher LLR values, indicating a higher probability of detecting true clusters. From this perspective, a *K*-value of 30 is ideal. However, to assess the effect of the *K*-value on the final results, we compared the results for *K* = 15 and *K* = 30. These results are presented in Table 3, and the spatial clusters are also presented on the map in Figure 3.

**Table 3:** Comparison of cluster results with different *K*-values in FleXScan.

2015 (*K* = 15)						2015 (*K* = 30)					
Level of clusters	Number of Sun-region	Number of cases	Population in risk	LLR	*p* - value	Level of clusters	Number of Sun-region	Number of cases	Population in risk	LLR	*p* - value
MLC	8	455	477,903	46.16	0.001	MLC	11	645	688,811	62.79	0.001
2	7	314	314,546	37.30	0.001	2	19	738	856,888	50.02	0.001
3	11	490	577,467	29.72	0.001	3	1	70	46,702	22.71	0.001
4	1	70	46,702	22.71	0.001	4	2	72	57,870	15.75	0.003
5	7	215	240,358	16.27	0.001	5	1	72	58,570	15.30	0.004
6	1	72	58,570	15.30	0.001	6	4	111	105,171	15.04	0.004
7	5	223	257,809	14.40	0.001	7	5	223	257,809	14.40	0.006

The results indicate that the spatial distribution of clusters is roughly the same when *K* = 15 and *K* = 30, but there are differences in cluster levels, as ordered by descending LLR values. Notably, there are variations in the spatial distribution of the MLC and the number of sub-regions included. When *K* = 30, the MLC contains more sub-regions and has a higher LLR value. Due to the limitation of the *K*-value, when *K* = 15, clusters 3 and 5 are identified as two separate clusters, with the LLR values of 29.72 and 16.27, respectively. However, these two clusters merge into a single, larger cluster when *K* = 30, with the LLR value increasing to 50.02.

### 3.4 Cluster visualization

To facilitate understanding of the results, we visualize the cluster analysis results on the map, using the color brightness to indicate the magnitude of the LLR statistical value. Darker colors correspond to higher LLR values, suggesting a greater likelihood of true clustering. Additionally, we employ different color lightness on the map to represent the incidence rates of townships and streets as a reference for the spatial clustering results.

## 4 Results

### 4.1 Comparison of SaTScan and FleXScan in evaluation metrics

Tables 4–8 present detailed comparison results of the SaTScan and FleXScan methods from 2015 to 2019. These tables include information on cluster level (ordered by descending LLR values), number of sub-regions (i.e., cluster size), number of cases, expected number of cases, population, RR value, LLR value, and *p* value.

**Table 4:** Comparison of SaTScan clusters and FleXScan clusters in 2015.

SaTScan-2015 (MSWS = 15%)								Restricted FleXScan-2015 (*K* = 30)							
Cluster level	Number of sub-regions	Number of cases	Expected cases	Population in risk	RR	LLR	*p* - value	Cluster level	Number of sub-regions	Number of cases	Expected cases	Populationin risk	RR	LLR	*p* - value
MLC	12	529	351.0	591,071	1.56	41.78	<0.0001	MLC	11	645	409.0	688,811	1.64	62.79	0.001
2	7	324	214.9	361,950	1.54	24.93	<0.0001	2	19	738	508.8	856,888	1.51	50.02	0.001
3	29	1,035	841.7	1,417,641	1.28	24.225	<0.0001	3	1	70	27.7	46,702	2.54	22.71	0.001
4	1	70	27.7	46,702	2.54	22.70	<0.0001	4	2	72	34.4	57,870	2.11	15.75	0.003
5	1	41	14.8	24,884	2.79	15.68	<0.0001	5	1	72	34.8	58,570	2.08	15.30	0.004
6	1	72	34.8	58,570	2.08	15.29	<0.0001	6	4	111	62.5	105,171	1.79	15.04	0.004
7	5	224	164.7	277,338	1.37	9.89	0.006	7	5	223	153.1	257,809	1.47	14.40	0.006

**Table 5:** Comparison of SaTScan clusters and FleXScan clusters in 2016.

SaTScan-2016 (MSWS = 10%)								Restricted FleXScan-2016 (*K* = 30)							
Cluster level	Number of sub-regions	Number of cases	Expected cases	Population in risk	RR	LLR	*p* - value	Cluster level	Number of sub-regions	Number of cases	Expected cases	Population in risk	RR	LLR	*p* - value
MLC	8	431	264.5	432,035	1.67	46.21	<0.0001	MLC	9	566	348.3	568,916	1.69	61.09	0.0005
2	1	91	36.4	59,445	2.52	29.02	<0.0001	2	6	388	227.5	371,545	1.75	48.79	0.0005
3	17	604	459.2	749,933	1.35	22.54	<0.0001	3	7	357	245.3	400,657	1.48	23.30	0.0005
4	3	169	109.2	178,290	1.56	14.31	<0.0001	4	4	200	135.9	221,932	1.49	13.53	0.01
5	1	59	27.8	45,394	2.13	13.28	0.0001	5	1	59	27.8	45,394	2.13	13.29	0.01
6	1	14	2.7	4,407	5.20	11.76	<0.0007	6	1	14	2.7	4,407	5.20	11.77	0.03
7	4	248	186.2	304,145	1.35	9.58	0.005								
8	1	63	35.8	58,518	1.77	8.44	0.01								
9	1	34	25.6	25,420	2.19	8.16	0.02								
10	1	52	29.0	47,400	1.80	7.39	0.03								

**Table 6:** Comparison of SaTScan clusters and FleXScan clusters in 2017.

SaTScan-2017 (MSWS = 20%)								Restricted FleXScan-2017 (*K* = 30)							
Cluster level	Number of sub-regions	Number of cases	Expected cases	Population in risk	RR	LLR	*p* - value	Cluster level	Number of sub-regions	Number of cases	Expected cases	Population in risk	RR	LLR	*p* - value
MLC	31	1,374	1048.8	1,829,721	1.40	56.64	<0.0001	MLC	20	1,126	768.4	1,340,576	1.57	85.03	0.0005
2	3	111	62.7	109,295	1.79	15.34	<0.0001	2	11	511	340.5	594,032	1.55	39.53	0.0005
3	4	215	145.1	253,094	1.50	15.07	<0.0001	3	4	188	128.4	223,975	1.48	12.40	0.02
4	1	64	33.9	59,207	1.90	10.61	<0.0001								
5	5	214	163.2	284,795	1.32	7.40	<0.0001								

**Table 7:** Comparison of SaTScan clusters and FleXScan clusters in 2018.

SaTScan-2018 (MSWS = 10%)								Restricted FleXScan-2018 (*K* = 30)							
Cluster level	Number of sub-regions	Number of cases	Expected cases	Population in risk	RR	LLR	*p* - value	Cluster level	Number of sub-regions	Number of cases	Expected cases	Population in risk	RR	LLR	*p* - value
MLC	12	614	441.7	815,615	1.44	32.70	<0.0001	MLC	13	703	495.0	913,950	1.48	42.75	0.0005
2	4	242	151.5	279,704	1.62	23.58	<0.0001	2	11	504	337.7	623,635	1.54	38.05	0.0005
3	3	111	60.2	111,182	1.86	17.33	<0.0001	3	10	509	355.2	655,898	1.47	31.51	0.0005
4	1	69	32.6	60,229	2.13	15.43	<0.0001	4	7	336	228.1	421,252	1.50	23.28	0.0005
5	13	586	485.2	895,986	1.23	10.76	0.002	5	3	213	150.2	277,388	1.43	11.95	0.03

**Table 8:** Comparison of SaTScan clusters and FleXScan clusters in 2019.

SaTScan-2019 (MSWS = 15%)								Restricted FleXScan-2019 (*K* = 30)							
Cluster level	Number of sub-regions	Number of cases	Expected cases	Population in risk	RR	LLR	*p* - value	Cluster level	Number of sub-regions	Number of cases	Expected cases	Population in risk	RR	LLR	*p* - value
MLC	6	364	227.5	422,571	1.64	36.26	<0.0001	MLC	12	706	427.6	794,267	1.74	82.93	0.0005
2	7	248	140.5	260,952	1.80	34.46	<0.0001	2	13	851	545.8	1,013,967	1.65	81.73	0.0005
3	7	329	205.4	381,515	1.64	32.77	<0.0001	3	8	362	238.8	443,597	1.55	28.78	0.0005
4	1	81	32.8	60,941	2.49	25.22	<0.0001	4	6	197	125.4	232,961	1.59	17.84	0.003
5	2	166	99.0	183,995	1.70	19.16	<0.0001	5	5	130	81.1	150,606	1.62	12.67	0.02
6	6	192	131.3	243,977	1.48	12.57	0.00048								
7	4	212	152.3	283,010	1.41	10.71	0.0026								
8	1	77	43.9	81,566	1.76	10.25	0.0039								
9	3	172	121.4	225,519	1.43	9.55	0.0073								

By comparing the relevant information of the two methods in Tables 4–8, particularly the LLR values and cluster sizes, we find that FleXScan identifies fewer clusters than SaTScan, but generally with higher LLR values. This suggests that the FleXScan method applies stricter criteria for defining clusters, reducing the likelihood of falsely identifying non-cluster areas as clusters. Consequently, while FleXScan may detect fewer true clusters, it is likely more accurate in identifying statistically significant clusters. The higher LLR values associated with FleXScan indicate stronger clustering signals, reflecting a greater probability of detecting true clusters.

In addition, we measure cluster size by the number of sub-regions covered. Tables 4–8 show that FleXScan identifies clusters with a larger average size but a smaller maximum size compared to the SaTScan method. This suggests that FleXScan tends to recognize larger, more consistent clusters but with a less extreme maximum size. In contrast, SaTScan produces results with greater variability in cluster sizes, indicating more dispersed and variable cluster sizes. Consequently, FleXScan demonstrates higher stability in cluster size compared to SaTScan, as it produces more consistent cluster sizes across different datasets.

Both methods generally produce MLCs of similar sizes, typically containing fewer than 16 sub-regions, representing less than 10% of the total 164 sub-regions. However, 2017 is an exception, with SaTScan’s MLC size reaching 31 sub-regions, compared to 20 sub-regions for FleXScan. This discrepancy is mainly due to SaTScan’s higher MSWS value of 20% in 2017, which was larger than in other years and resulted in a larger MLC size.

This difference in performance arises from FleXScan’s use of an exhaustive algorithm, which evaluates all potential scan windows to pinpoint those with the highest LLR values. This approach allows FleXScan to be more precise in detecting clusters and identifying significant clustering patterns, as it thoroughly assesses a wide range of possible cluster configurations. By contrast, SaTScan utilizes a circular scanning window, which can constrain its ability to capture irregularly shaped or more complex clustering patterns. The circular window’s limitations can result in less accurate cluster detection and higher variability in the sizes of detected clusters. Furthermore, the SaTScan method, which involves scanning regions with progressively larger circles, might miss clusters that are not well-aligned with the circular shape or that have non-uniform spatial distributions. This can lead to less consistent results and a greater variability in cluster sizes, as observed in the data.

### 4.2 Comparison of SaTScan and FleXScan in cluster visualization

**Figure 4:**
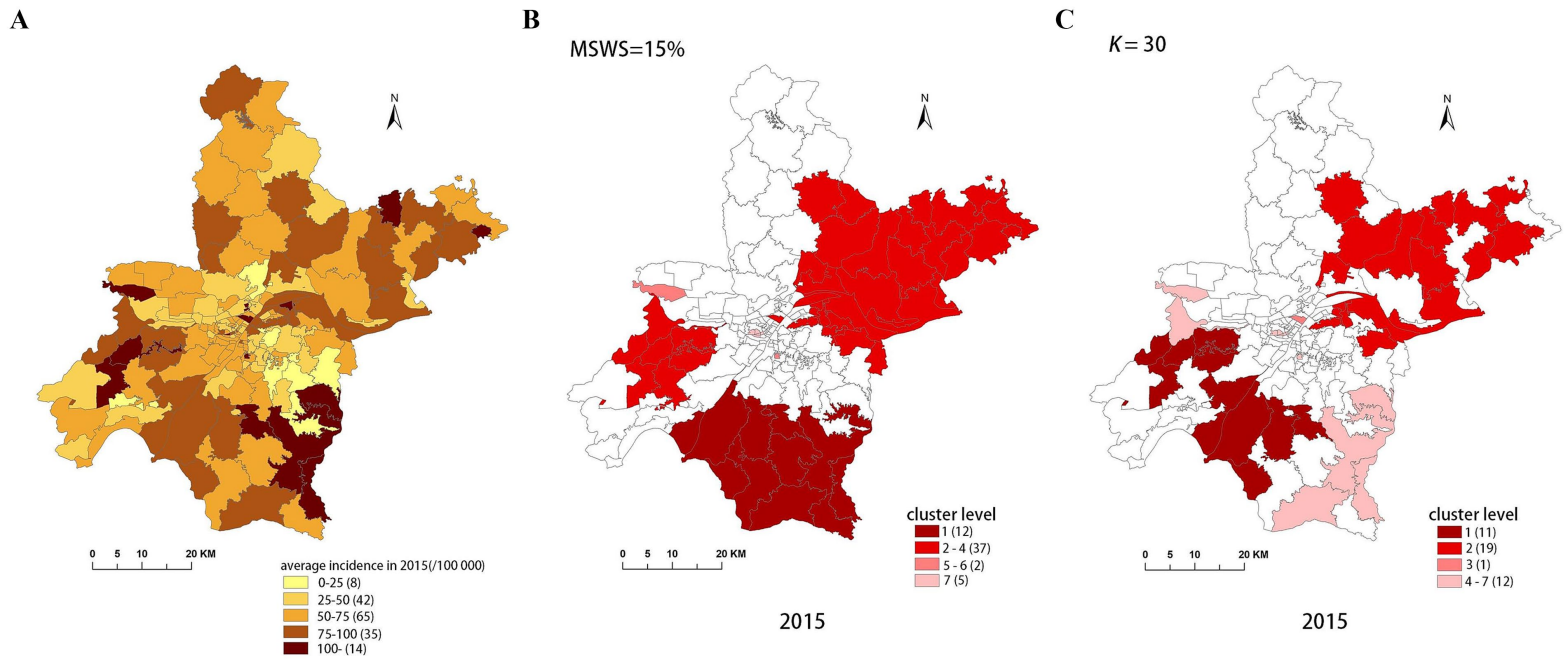
Spatial distribution map of tuberculosis in Wuhan in 2015. **(A)** The annual incidence map, **(B)** the SaTScan clusters result map, **(C)** the FleXScan clusters result map.

**Figure 5:**
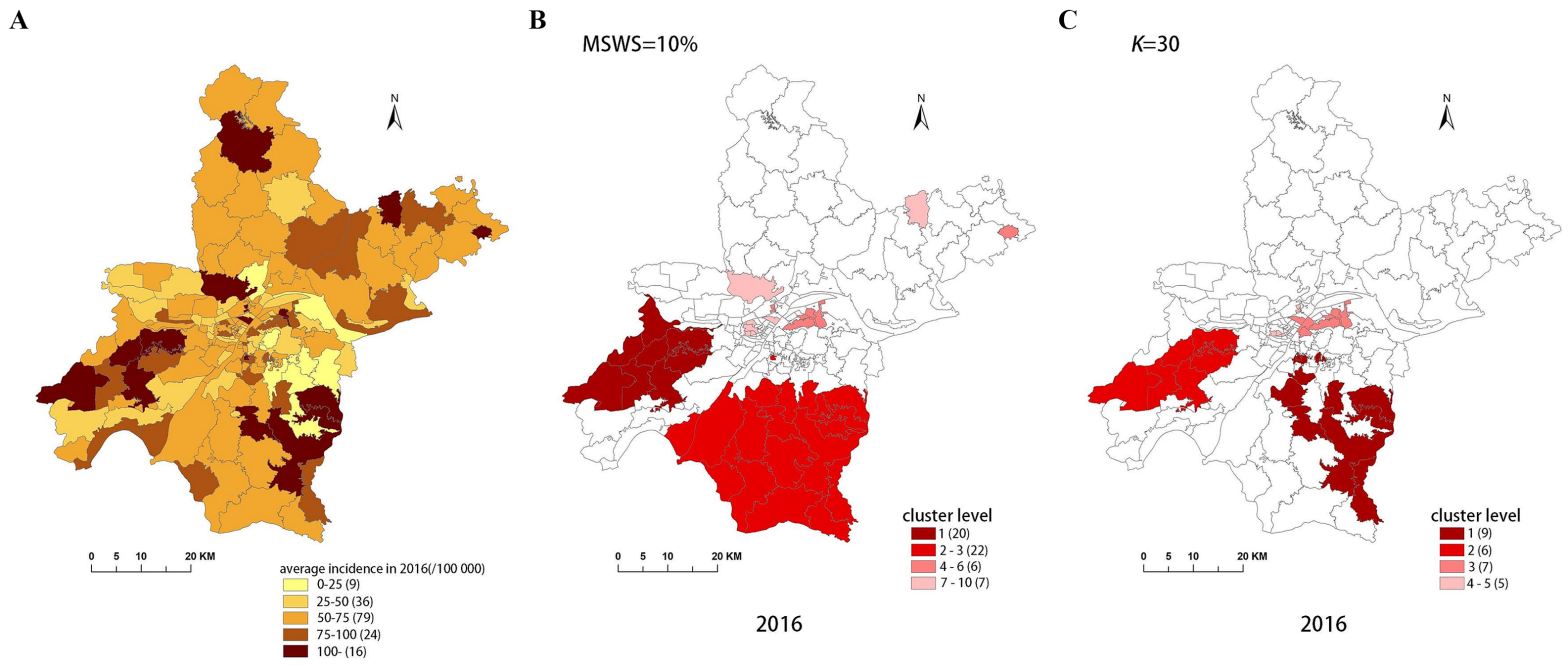
Spatial distribution map of tuberculosis in Wuhan in 2016. **(A)** The annual incidence map, **(B)** the SaTScan clusters result map, **(C)** the FleXScan clusters result map.

**Figure 6:**
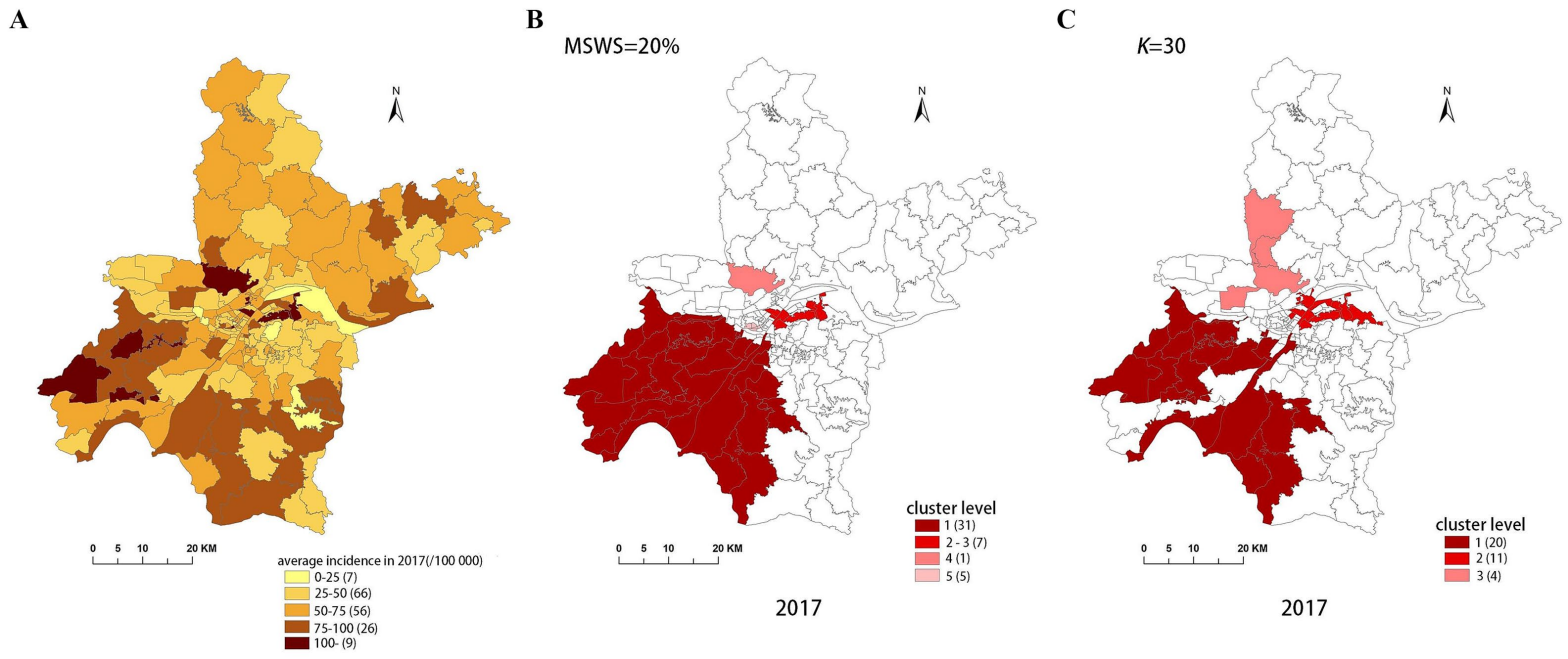
Spatial distribution map of tuberculosis in Wuhan in 2017. **(A)** The annual incidence map, **(B)** the SaTScan clusters result map, **(C)** the FleXScan clusters result map.

**Figure 7:**
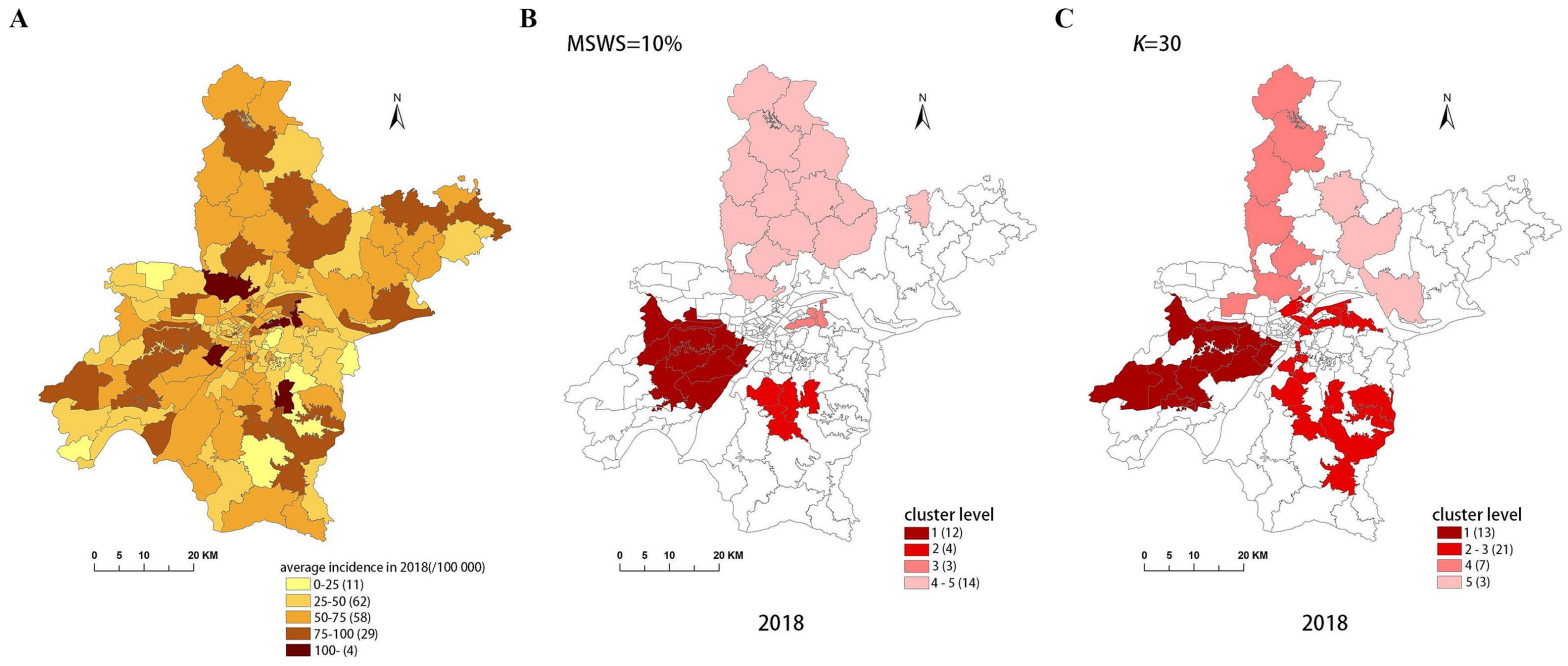
Spatial distribution map of tuberculosis in Wuhan in 2018. **(A)** The annual incidence map, **(B)** the SaTScan clusters result map, **(C)** the FleXScan clusters result map.

**Figure 8:**
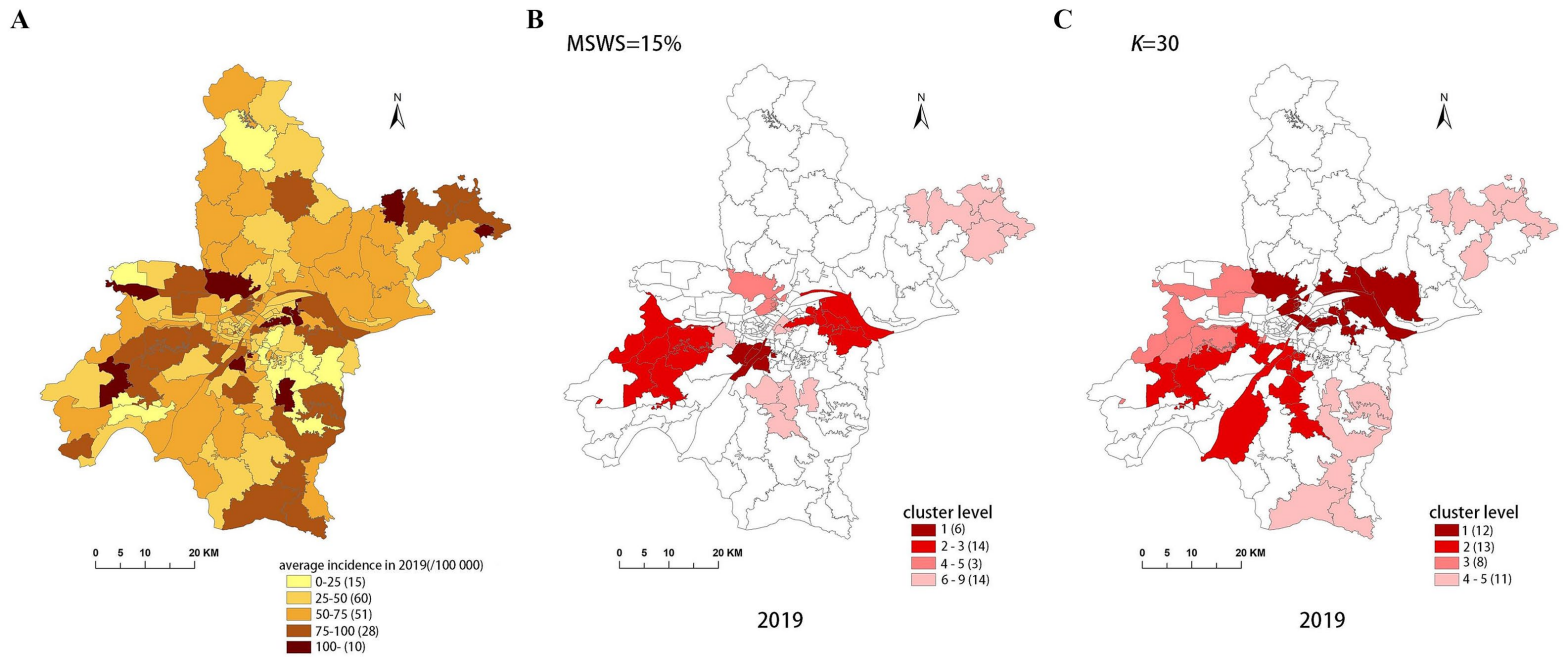
Spatial distribution map of tuberculosis in Wuhan in 2019. **(A)** The annual incidence map, **(B)** the SaTScan clusters result map, **(C)** the FleXScan clusters result map.

Figures 4–8 show the clustering results generated by the SaTScan and FleXScan methods in Wuhan from 2015 to 2019 on maps. Additionally, pulmonary tuberculosis incidence maps are provided for comparison and reference.

To accurately represent the cluster areas, we used the cluster regions comprising all polygons whose centroids are enclosed by the cluster circle, rather than directly using the cluster circles generated by SaTScan. Furthermore, since certain years have numerous cluster levels with only a few regions per level (e.g., in 2016, SaTScan identified a total of 10 cluster levels, many of which included only one sub-region). To improve the legibility of the visualization, we categorized the original clustering regions into four categories based on LLR values and the number of included sub-regions. The legend specifies the number of sub-regions in each category, and the MLC was assigned a single distinct category. This classification converted the original data into ordinal data, represented by different lightness of color in Figures 4–8. The color intensity in each map corresponds to the clustering area level determined by the LLR value, with darker colors indicating higher likelihoods.

From Figures 4–8, it is evident that the clusters obtained by SaTScan are more regular in shape, whereas those identified by FleXScan exhibit excessive irregularity. Clusters with highly irregular shapes may be less meaningful, as they complicate the assessment of geographical significance for practitioners (9, 17).

Overall, the clusters identified by SaTScan and FleXScan generally align with the spatial distribution of high-incidence areas, primarily located in the suburban districts of Wuhan, which are sparsely populated and economically underdeveloped. However, some high-incidence areas were not identified as clusters, suggesting that the elevated incidence rates in these regions may be random. Although the spatial coverage of clusters identified by both methods is largely similar, there are significant differences in the LLR values and the specific sub-regions included. Notably, the spatial distribution of MLCs identified by the two methods from 2015 to 2019 differs considerably, with the exception of 2017, where the distributions are similar.

## 5 Discussion

In this study, we undertook a detailed comparative analysis of the SaTScan and FleXScan methods for disease clustering using real data from 2015 to 2019. This comparison aimed to explore the strengths and limitations of each method in accurately detecting disease clusters.

### 5.1 Real disease data

Unlike most previous studies that relied on simulated data, our research utilized real disease data. Although this limited our ability to use common quantitative metrics, such as sensitivity, specificity, PPV, and YDI, to determine the optimal parameter settings and compare method performance, using LLR values and cluster size as our analysis metrics is still appropriate, albeit not entirely comprehensive. Nevertheless, real disease data better reflect actual disease distribution and trends, enhancing the realism and generalizability of our results and providing more reliable support for public health decision-making.

### 5.2 Parameter settings

The Gini coefficient, traditionally used to determine the MSWS in SaTScan, has been validated as effective by some researchers (17, 18). However, our findings align with those of Li et al. (15), who identified limitations in this approach. Specifically, the Gini coefficient measures overall distribution uniformity across the entire region and may not capture the nuances of smaller, individual clusters when multiple clusters are present. This may lead to multiple small clusters being combined into one large cluster, resulting in distorted results, as confirmed in our study illustrated in Section 3.2. To address this issue, we recommend combining the Gini coefficient approach with a gradual reduction in the threshold to accurately identify and separate individual clusters, thereby obtaining more reliable clustering results.

FleXScan identifies clusters using LLR values. While setting *K* = 30 is theoretically optimal, our study reveals that focusing solely on the highest LLR values can lead to clusters with highly irregular shapes (21–23). Duczmal et al. (21, 22) have noted that such irregular shapes can complicate geographic interpretation and suggest that both LLR values and cluster shapes should be considered together to achieve clusters that are both statistically significant and meaningful. Irregular cluster shapes may arise from specific geographic features, population distribution, or data noise and might not accurately reflect the actual disease distribution. Therefore, considering the regularity of cluster shapes is important to avoid misleading interpretations and ineffective public health interventions. The current version of FleXScan lacks features to control or modify cluster shapes, highlighting the need for further developments in this area.

### 5.3 Computational efficiency

Although both methods can be implemented through software, it is essential to discuss their computational efficiency to gain a deeper understanding of the differences in their results. The efficiency primarily depends on the number of scanning windows that need to be calculated.

In the SaTScan method, let the entire study area contain *m* sub-regions. For each region, the scanning radius varies systematically from 0 to a predefined maximum (MSWS value), centered on each region. If each region has *T* concentric circular windows, the maximum number of windows that need to be calculated is *m* × *T*.

In contrast, the FlexScan method requires calculating a greater number of scanning windows. The process is as follows:

Let $Z_{i}$ represent region *i* (1 ≤ *i* ≤ *m*), and $Z_{ik}$ denote the scanning window formed by sub-region $Z_{i}$ and its ***k*-1** connected neighboring sub-regions. The basic method for determining these *k*-1 sub-regions is:

1. Calculate the *K*-1 nearest neighboring sub-regions of $Z_{i}$ (which may not necessarily be adjacent to $Z_{i}$ ).
2. From these *K*-1 neighboring sub-regions, select *k*-1 (noting that 1 ≤ *k* ≤ *K*) while ensuring that they form a “connected” scanning window with $Z_{i}$ .

For example, with *k* = 4, this means that the scanning window consists of $Z_{i}$ and three neighboring sub-regions. In the worstcase, the selection of these three sub-regions can result in $C_{K-1}^{3}$ combinations. Therefore, theoretically, the FleXScan method may need to calculate $m*2^{K-1}$ windows in the worst case. Although the requirement for “connectivity” among sub-regions means that the actual number of scanning windows calculated will be lower, it remains substantial. This is why the FleXScan software typically recommends that the value of *K* should not exceed 30, with a default value of 15.

From a computational efficiency perspective, the SaTScan method demonstrates higher efficiency, while the FleXScan method is comparatively less efficient. Thus, enhancing the computational efficiency of the FleXScan method presents a valuable area for further research. Both classic methods can currently be implemented directly through software, allowing researchers to focus less on their computational efficiency. However, any optimizations or improvements based on these methods must inevitably consider computational efficiency.

### 5.4 Result visualizations

This study employed a map visualization method to display the spatial distribution of disease clusters, using color brightness to indicate risk levels. However, differences in cluster distributions from the SaTScan and FleXScan methods are not intuitively discernible. Introducing interactive visualization tools would enhance the comparison of distribution differences among clusters with varying risk levels, improving the clarity and practicality of the analysis.

### 5.5 Limitations

Despite the in-depth comparison and analysis of the SaTScan and FleXScan methods, our study has several limitations:

1. Before applying FleXScan, obtaining a complete spatial adjacency matrix for the specific geographic area is crucial. Missing spatial adjacency relationships can bias clustering results, making preliminary topological checks essential to ensure the integrity of the adjacency matrix. In this study, we defined the spatial adjacency matrix using queen adjacency, which considers shared vertex connections. This may explain the irregular cluster shapes produced by FleXScan. Since queen adjacency only considers regions sharing a vertex as neighbors, it may lack precision, especially for irregular or complex cluster shapes. To enhance the accuracy and interpretability of clustering results, future research could explore alternative adjacency definitions, such as rock adjacency (shared edge adjacency) or bishop adjacency (considering both shared vertices and edges).
2. Although our study used multi-year disease data, the cross-sectional nature of the data limited the use of space–time scan statistics, restricting a full assessment of SaTScan and FleXScan’s spatiotemporal sensitivity and precision. Additionally, our analysis was limited to Wuhan City and did not include data from broader scales like Hubei Province. Future research should assess these methods across different geographical scales, such as provincial or national levels, to provide a more comprehensive evaluation and increase the generalizability of the results.
3. Our comparison focused on circular and flexible-shaped scan windows. However, elliptical scan windows, which can adjust their radii in two directions to better fit non-uniform spatial distributions (6), warrant further exploration and evaluation in future research.

## 6 Conclusion

In this study, we concentrated on determining the optimal parameter settings for circular and flexible-shaped scan statistics and their effects on clustering results. We also explored the characteristics of these two methods and the influence of different scan window shapes on accuracy and reliability, offering valuable insights for future research.

While the FleXScan method may offer advantages in terms of result accuracy, disease spatial clustering patterns are highly complex. To mitigate the limitations of a single method, it is advisable to use a combination of methods to determine the final clustering results. Furthermore, the exploration of disease spatial clustering characteristics should be integrated with the study of influencing factors. Investigating clustering patterns not only aids in developing more effective prevention and control strategies but also reveals the factors and dynamics that influence disease occurrence and spread. By integrating these research methods, a more comprehensive understanding of disease transmission and its impact can be achieved, leading to more targeted and effective intervention measures.

## Data Availability

The original contributions presented in the study are included in the article/supplementary material, further inquiries can be directed to the corresponding author.
